# Experimental evidence of long-term oceanic circulation reversals without wind influence in the North Ionian Sea

**DOI:** 10.1038/s41598-020-57862-6

**Published:** 2020-02-05

**Authors:** Angelo Rubino, Miroslav Gačić, Manuel Bensi, Vedrana Kovačević, Vlado Malačič, Milena Menna, Maria Eletta Negretti, Joël Sommeria, Davide Zanchettin, Ricardo V. Barreto, Laura Ursella, Vanessa Cardin, Giuseppe Civitarese, Mirko Orlić, Boris Petelin, Giuseppe Siena

**Affiliations:** 1 University Ca’ Foscari of Venice, Dept. of Environmental Sciences, Informatics and Statistics, Via Torino 155, 30172 Mestre, Italy; 2 Istituto Nazionale di Oceanografia e di Geofisica Sperimentale - OGS, Borgo Grotta Gigante 42/C – 34010, Sgonico (TS), Italy; 3 National Institute of Biology, Marine Biology Station, Piran, Fornače 41, 6330 Piran, Slovenia; 4 LEGI, CNRS UMR5519, University of Grenoble Alpes, Grenoble, France; 5 Andrija Mohorovičić Geophysical Institute, Faculty of Science, University of Zagreb, Horvatovac 95, 10000 Zagreb, Croatia

**Keywords:** Physical oceanography, Climate and Earth system modelling

## Abstract

Under the emerging features of interannual-to-decadal ocean variability, the periodical reversals of the North Ionian Gyre (NIG), driven mostly by the mechanism named Adriatic-Ionian Bimodal Oscillating System (BiOS), are known as impacting on marine physics and biogeochemistry and potentially influencing short-term regional climate predictability in the Eastern Mediterranean. Whilst it has been suggested that local wind forcing cannot explain such variability, aspects of the alternative hypothesis indicating that NIG reversals mainly arises from an internal ocean feedback mechanism alone remain largely debated. Here we demonstrate, using the results of physical experiments, performed in the world’s largest rotating tank and numerical simulations, that the main observed feature of BiOS, i.e., the switch of polarity of the near-surface circulation in the NIG, can be induced by a mere injection of dense water on a sloping bottom. Hence, BiOS is a truly oceanic mode of variability and abrupt polarity changes in circulation can arise solely from extreme dense water formation events.

## Introduction

Natural laboratory basins are regions of the world ocean in which many of the physical and biogeochemical phenomena characterizing the global ocean occur on substantially smaller spatial and temporal scales^1–5^.

Modes of variability in such miniature oceans are of special interest: understanding their emergent spatio-temporal features in relation to the basin’s characteristics can disclose a host of processes and relations of paramount importance for the comprehension of the world ocean functioning and evolution. Especially when such modes connect surface, interior and deep ocean, they can be pivotal in entangling the intricate interactions involved in the basin’s long-term variability and in paving the way to an increased near-term predictability of interior dynamics^6^.

The circulation of the upper layer of the North Ionian Gyre (NIG), in the Eastern Mediterranean, shows periodic reversals^7–10^ that have been demonstrated to be associated with major variations in physical and biogeochemical properties of the local water masses, with potential impacts on the broad marine (eco)system functioning^11–13^.

Observations indicate that this phenomenon, baptized Adriatic-Ionian Bimodal Oscillating System (BiOS), generally occurs about every five years, although a remarkable irregularity in the timing of phase reversals has been observed as well^14^.

**Figure 1:**
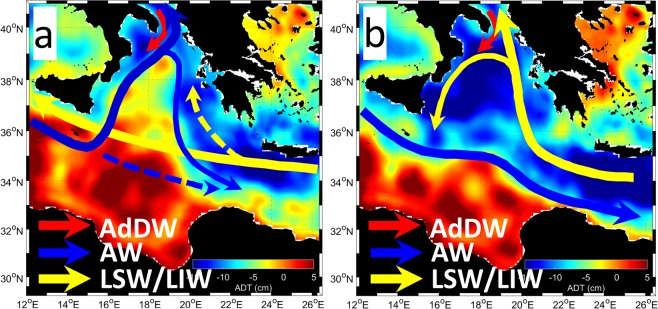
Schematic representation of the geostrophic circulation in the North Ionian Gyre region. (**a**) anticyclonic phase. (**b**) cyclonic phase. The background maps are annual averages of Absolute Dynamic Topography (ADT) in 1996 and 2003, respectively. For the water mass acronyms see text. Adapted from Menna *et al*.^35^.

Three alternative explanations of the NIG reversals have been proposed. The first one attributes the NIG reversals mainly to local wind forcing^15,16^; accordingly, the irregular alternation of BiOS phases is simply explained by the stochastic nature of the local mesoscale atmospheric variability. The second explanation includes the BiOS complex feedback mechanism encompassing near-surface, intermediate and dense, convectively generated, waters (i.e., respectively, Atlantic Water, AW, Levantine Intermediate Water, LIW, and Adriatic Dense Water, AdDW). Additionally, Theocharis *et al*.^17^ and Velaoras *et al*.^18^ have hypothesized the existence of an internal thermohaline pumping mechanism taking into account the whole upper thermohaline cell of the Eastern Mediterranean, which regulates the salinity distribution and the dense water formation processes in the Adriatic and Aegean basins. According to the different circulation phases of the NIG, different water masses are advected into the Adriatic. The relatively fresher, hence lighter AW dominates during the anticyclonic phase, while the relatively saltier, hence heavier LIW dominates during the cyclonic phase (Fig. 1). The predominance of either AW or LIW in the neighboring Adriatic shapes the preconditioning to the local winter convection, which determines the density of the newly formed AdDW spreading toward the Ionian abyss^3,19,20^. From such density field a horizontal pressure gradient emerges which contrasts the dominating near-surface circulation and triggers its reversal with the corresponding water mass transport toward the Adriatic. According to this mechanism, the deep ocean circulation in the Ionian Sea sets the typical time scale of intrinsic BiOS variability, upon which an irregular component, linked to major, episodic events of copious deep water (mostly AdDW) formation can be superimposed^21^. Such an event has been recorded in 2012, when an extremely harsh winter over the Adriatic area caused the production of a large amount of very dense North Adriatic Dense Water (NAdDW)^22,23^ that contributed to enhance the AdDW outflow^14^.

It should be noted that deep water formation in the Adriatic Sea has experienced dramatic changes under past climate conditions^24^. Moreover, the area is subjected to broader climate shifts, encompassing the replacement of waters of Adriatic origin by waters of Aegean origin in the Ionian abyss, like, e.g., the so-called Eastern Mediterranean Transient (EMT)^25^. Recent studies have presented evidence on occurrences of other EMT-like phenomena in the last centuries^6,26^.

The alternative explanations of the NIG reversals mentioned above do not only identify different causes for the observed phenomenon. More importantly, they depict the NIG reversals as possessing different nature and scientific relevance: either being a basic wind-induced circulation or, instead, representing a proper mode of oceanic variability. Note that phenomena from which such alternative behavior could emerge have been observed also in other regions of the World Ocean, which puts our investigation in a context of more general relevance^27–29^. A key step forward in our understanding of the BiOS, particularly towards establishing it as a mode of variability, regards thus whether interior ocean processes alone are capable of triggering reversals in the NIG circulation. For this purpose, realistic numerical models do not offer yet a detailed description of the functioning of the Adriatic/Ionian and Levantine system, as they are still unable of accurately describing the complete net of intricate spatial and temporal oceanic/atmospheric relations responsible for the observed variability.

Given these premises, and based on the consideration that, in an idealized context, the northern part of the Ionian basin behaves as a two-layer system (having AW, LIW and light AdDW as upper layer, and dense AdDW as lower layer), the BiOS-CRoPEx (Adriatic-Ionian Bimodal Oscillating System – Coriolis Rotating Platform Experiment) tank experiments were designed to demonstrate the capability of internal processes alone to dictate the circulation in the upper thermocline. The main experiment we will discuss here (“EXP27”), in particular, is inspired by the winter 2012 convective episode in the Adriatic basin. We will however demonstrate that a major part of the observed dynamics arises also for other experimental configurations. The use of a layered numerical model solving the unsteady, nonlinear shallow-water equations for water masses possessing near-surface as well as intermediate and bottom-arrested frontal features ensures robustness of the obtained experimental evidences against crucial parameter variations shaping the major dynamical features involved in the phenomenon.

## Results

Winter 2012 was characterized by extremely harsh weather conditions over the Mediterranean region causing the formation of a particularly abundant dense water in the Adriatic^22,30^. During the following spring season, these newly formed waters spread into the Ionian Sea, simultaneously with the occurrence of a circulation reversal of the NIG^14^. Remote sensing as well as *in situ* oceanographic measurements provide a rather accurate characterization of the surface and subsurface anomalies that constituted the 2012 event^14^. However, controlled experiments in a simplified framework remain to be performed in order to demonstrate, beyond any doubts, that an exclusive causal mechanism exists between spreading of dense Adriatic bottom waters into the Ionian and NIG reversals.

**Figure 2:**
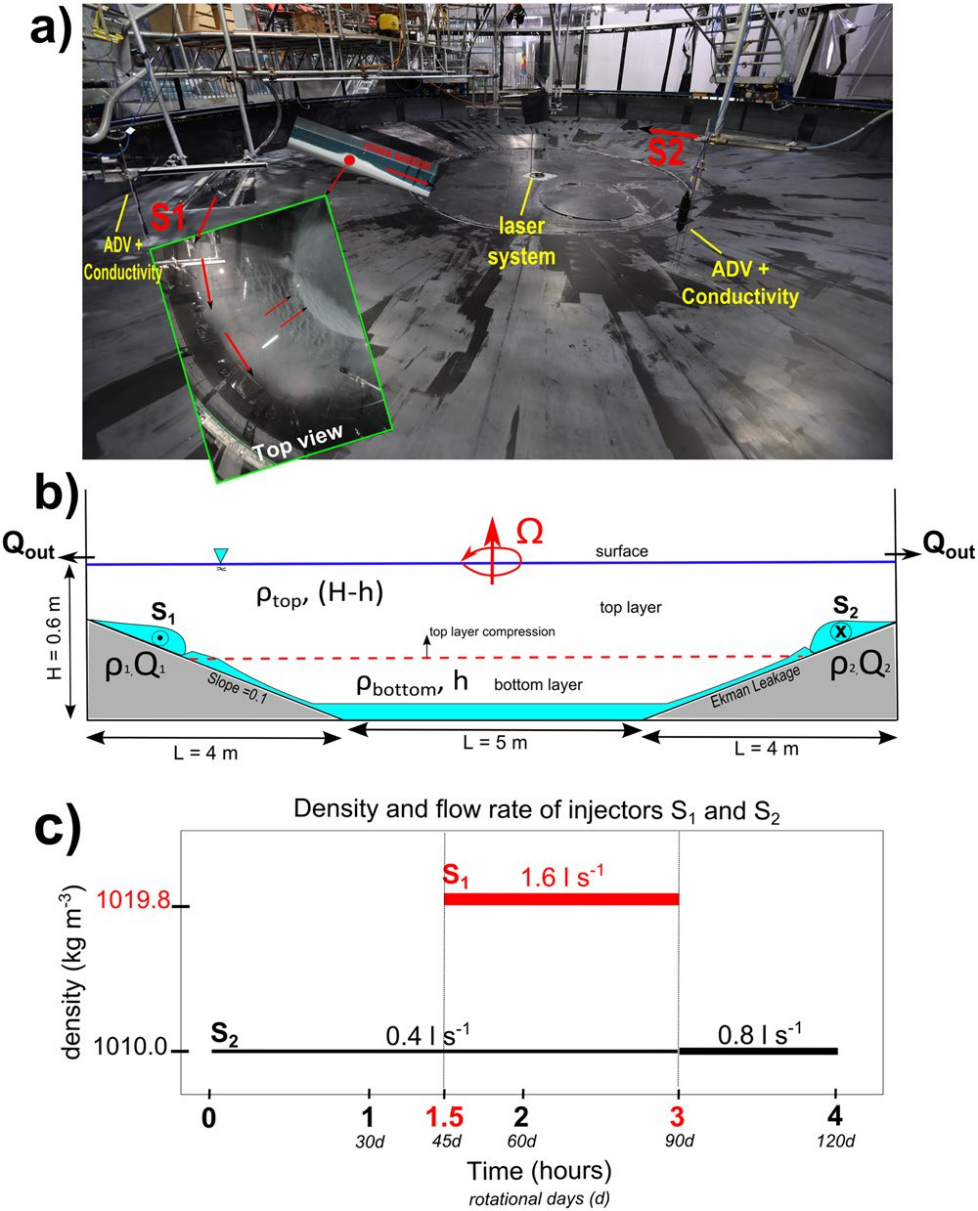
Laboratory experimental setup. (**a**) photo of the LEGI tank prepared for the BiOS-CRoPEx experiment. (**b**) Schematic vertical cross section of the tank. The turquoise layer represents the dense water injected in the two-layer system (densities ρ_top_ and ρ_bottom_) through the injectors S_1_ (density ρ_1_ and discharge Q_1_) and S_2_ (density ρ_2_ and discharge Q_2_, see methods for full description of symbols). (**c**) Timeline of the injection during the experiment. The solid lines represent the injected water densities and the duration of injections, with discharge rate reported above each segment of the experiment.

The series of BiOS-CRoPEx experiments were conducted at the LEGI Coriolis tank under the hypothesis that the behaviour of the Adriatic/Ionian basin can be described accurately by a two-layer system, at least as far as the BiOS mechanism and NIG reversals are concerned (Fig. 2, see also methods).

Two sources of dense water were implemented (see Fig. 2a,b): the S_2_ to represent the background intermediate Adriatic water leaving the basin toward the Ionian Sea; the S_1_ to provide the very dense Adriatic water, mimicking convectively produced water mass during winter 2012. The source S_2_ was active from the beginning of the experiment, while the source S_1_ was active from the 45^th^ until the 90^th^ experimental day (Fig. 2c). The background lower density Adriatic flow, which had remained switched on throughout the experiment, was doubled in a flow rate after the dense water switch off. This to represent the fact that the copious convective activity caused, for continuity, a large flow of near-surface water into the Adriatic which, eventually, had to leave the basin as intermediate current after the end of the convection.

**Figure 3:**
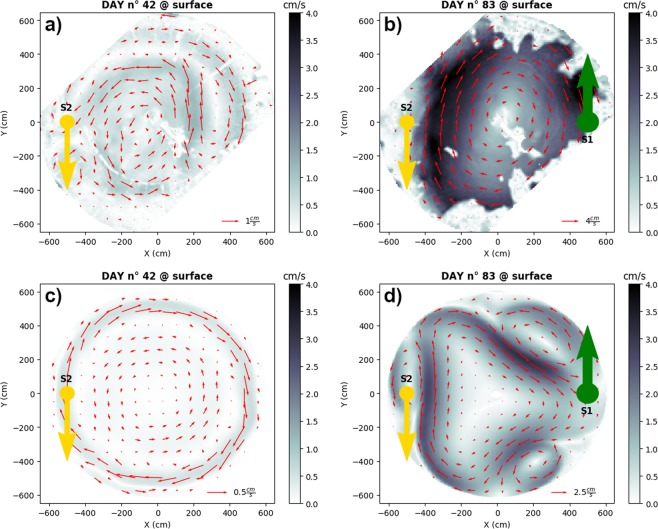
Velocity field observed during the BiOS-CRoPEx experiment. (**a**) Horizontal distributions of the flow (arrows) and velocity magnitudes (shading) in the uppermost level at experimental day 42, before the dense water injection. (**b**) Same as (**a**) but at experimental day 83, before the end of the dense water injection. (**c**) Same as (**a**), but simulated by the numerical frontal model. (**d**) Same as (**b)** but simulated by the numerical frontal model. S_1_ and S_2_ are the sources of dense water.

The velocity field produced experimentally in the upper layer rapidly responds to the dense water injection (Fig. 3a,b): the area of almost homogeneous cyclonic vorticity present in the initial experimental phase due to the injection of lighter water (see Fig. 3a) evolves toward a strong coherent basin-wide anticyclonic vortex just about ten days after the beginning of the dense water injection (i.e., around experimental day 55) and clearly established on day 83 (Fig. 3b). The numerical model is capable of reproducing accurately such dynamics, including the emergence of mesoscale vortices of opposed polarity (Fig. 3c,d) and helps elucidating its variability in details. Obviously, numerical models cannot replicate exactly the timing of such submescoscale activity. In the experiments, its variability depends intricately on tank geometry, flow rate and density contrast^31,32^. In the simulations it is, for a certain model configuration, very sensible on different parameters, such as eddy viscosity as well as interface and bottom friction coefficients.

**Figure 4:**
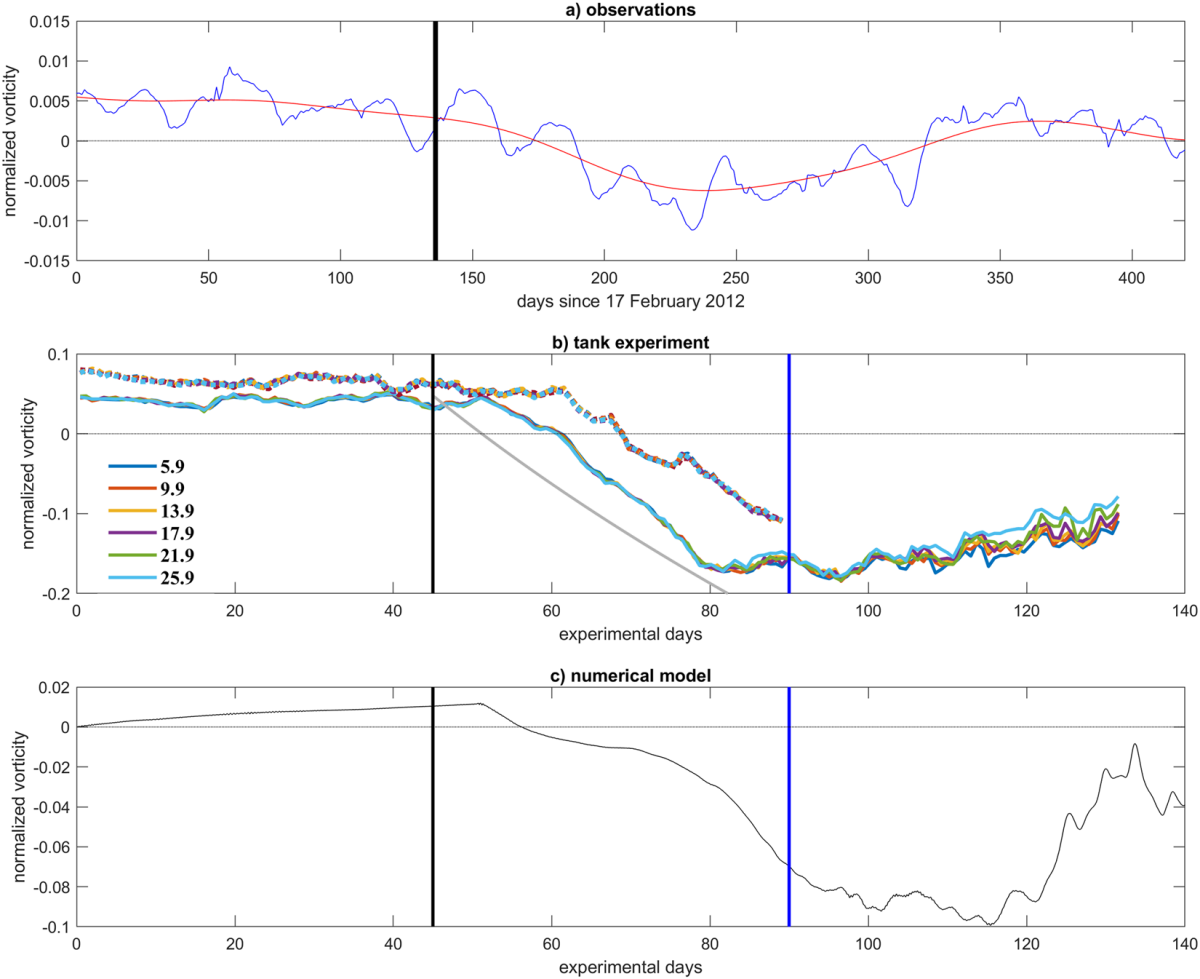
Normalized vorticity (vorticity divided by the Coriolis parameter (10^−4^ s^−1^, 10^−1^ s^−1^, 10^−1^ s^−1^ for panels a, b, and c, respectively). (**a)** Surface geostrophic vorticity observed in the Northern Ionian area during the year 2012. **(b)** Spatially averaged vorticity field in the upper layer within the central portion of the laboratory tank for two different experiments: Solid lines represent the normalized vorticity of EXP27 at different depths (in cm) from the free surface, dotted colored lines represent the same normalized vorticities for EXP25, similar to EXP27, but with an injection of dense water having density ρ = 1019.5 kg/m^3^ at day 57 and with a larger flow rate (2l/s). **(c)** Near-surface spatially averaged vorticity field within the central portion of the tank as simulated by the numerical frontal model. The vertical lines indicate the onset and the end of the dense-water injection. Please note the different temporal scales in the abscissae between oceanic conditions on one hand and, physical and numerical models on the other. Grey line in c represents the vorticity change computed from the theoretical expression (see Methods).

From the tank vorticity evolution (Fig. 4b) it is evident that after the cyclonic phase has established, a rapid tendency toward a circulation reversal occurs within a couple of days after the onset of the dense water injection. The anticyclonic phase is established about twenty days later and intensifies progressively until reaching a peak around experimental day 90. Note that potential vorticity conservation (Fig. 4b, grey line) alone can predict the observed transition.

The experiments were designed based on the exceptional hydrographic conditions observed in the Adriatic/Ionian system during winter and spring 2012 due to the effect of a severe weather over the Mediterranean region, and in particular over the Italian peninsula. Indeed, different aspects of the observed dynamics are captured by the laboratory experiment and by the corresponding numerical simulations. Figure 4a illustrates the observed NIG evolution as presented by geostrophic vorticity from altimetric data. A cyclonic circulation existed in winter 2012, which stems from the cyclonic phase of the BiOS established in 2011^14^ and was expected to persist for several years according to the internal BiOS mechanism. However, in late spring 2012, in correspondence with the arrival in the deep regions of the Ionian basin of convectively generated very dense AdDW produced during the previous winter, a switch toward anticyclonic circulation in the NIG region occurred, characterized by a peak anticyclonic vorticity in late summer 2012 and a return to prevailing cyclonic conditions in the following winter.

The tank experiments captured the observed evolution. In Fig. 4b the spatially averaged vorticity field in the upper layer within the central portion of the laboratory tank is shown for two different experiments: Solid lines represent the normalized vorticity of EXP27 at different depths (in cm) from the free surface, dotted colored lines represent the same normalized vorticities for EXP25, similar to EXP27, but with an injection of dense water having density ρ = 1019.5 kg/m^3^) at day 57 and with a larger flow rate (2 l/s). Both evolutions clearly show a reversal of the vorticity, which is therefore induced merely by the internal dynamics. In particular, the physical processes responsible for such reversal can be sketched as follows: The dense water discharge on the slope results in the currents flowing downslope, which takes place at distances on the order of the Rossby radius of deformation from the source. At these distances from the source the viscous effects can be considered negligible. On the other hand, at larger length scales the viscous draining becomes prevalent. The flow turns along-slope and the main mechanism draining the dense water and causing the stretching of the water column is in fact viscous draining^31,32^. Therefore, during the entire process of the dense water discharge, a continuous downslope flow takes place causing the stretching of the water column on the slope and the squeezing of the surface layer in the flat-bottom central area. Consequently, a continuous production of the anticyclonic vorticity in the upper layer of the central area takes place, while in the slope area the cyclonic vorticity is generated in the form of cyclonic eddies or in a spatially continuous cyclonic shear.

In the numerical simulation a similar situation is produced (Fig. 4c): the activation and stopping of dense-water injection mark three circulation phases: an initial cyclonic phase, a change in polarity induced by the injection of dense water peaking in an anticyclonic vortex, and a tendency toward a reestablishment of the initial cyclonic state after the dense-water injection stops. Note that the durations of the two transitions (cyclonic to anticyclonic and vice versa) substantially differ between numerical simulation and ocean, being the timescale of the simulated event about half of the observed one. Such discrepancy is to be expected since it arises from the distortions induced in the system by the simplified bathymetry and volume constraints. If, for instance, we consider the ratio between inertial period and residence time in the experiment as well as in the real ocean, we can see that such non-dimensional parameter is approximately twice as large in the ocean than in the tank experiment. Therefore, a factor of two or so appears in the time needed for the inversion of vorticity to be established in the two cases.

## Conclusions

In this paper, we analyzed results from observations, laboratory, and numerical experiments designed to tackle one of the major unanswered questions regarding ocean dynamics in the Mediterranean Sea, namely whether the observed periodic inversion of the NIG circulation can be attributed solely to interior ocean dynamics. Our results allow to establish the main role of the Adriatic/Ionian BiOS as an internal mode of oceanic variability sensitive to remote atmospheric forcing able to induce dense water formation, as exemplified by the 2012 BiOS fluctuation. As such dynamics interact with the thermohaline conveyor belt of the Eastern Mediterranean, shaping and being shaped by the deep convection in the Adriatic basin, a deeper understanding of the characteristic timescales and feedback mechanisms involved in the BiOS would pave the way toward improved short-term regional climate predictions in the Eastern Mediterranean, hence yield valuable information to be translated from such miniature ocean to a larger-scale context.

## Methods

### Facility and experimental design

The Coriolis rotating platform at LEGI in Grenoble (France) consists of a circular tank of 13 m diameter and 1 m depth. It is hence the World’s largest rotating tank for geophysical investigations (see www.legi.grenoble-inp.fr/). In such a tank, different bathymetries and measurement configurations can be established. In our case, a slope s = 0.1 was realized using an axisymmetric conical-shaped bottom descending toward the center of the tank. The inclined surface descended from the tank edge at a height of 40 cm, thus yielding a length of 4 m and leaving the portion of the tank within a radius of 2.5 m from the center with a constant depth.

The tank, filled with two layers of water with different densities, was put on solid-body anticlockwise (cyclonic) rotation. Particular attention was paid to the filling, which lasted several hours in order to ensure a sharp interface between the two layers. Saline solutions were then injected through two injectors, namely S_1_ and S_2_ (see Fig. 2a), each made of a tube ending on a rigid plate covered by a flexible plastic cover, fixed at the two sides to the bottom plate 1 m distant from the top of the slope area. This configuration enables the saline current to attain an almost geostrophic equilibrium within the tube without mixing with the fresh ambient water during the critical adjustment phase. The dimensions of the flow (L, h_j_) and the flow rate Q = uh_j_L were estimated prior to the experiment to produce velocities next to the theoretical along-slope speed u_N_ = g’ s/f characterizing a geostrophically balanced bottom-arrested current (Nof speed^33^). In the previous formula f = 4π/T, with T the tank rotation period and g’ the reduced gravity of the two-layer system. With this balance, we obtain a flow width of the density current L ⋍ 50 cm and a maximum height h_j_ ⋍ 2 cm at the mouth of the injectors (Fig. 2). A pump with a maximum capacity of 4 l s^−1^ was used to maintain the flow rate within the range between 0.4 and 1.6 l s^−1^. Outlets were represented by a thin gap of a few millimeters at the top of the slope around the circumference of the tank, to allow for discharging the fresh water and keep the total water depth constant throughout the experiment, simultaneously ensuring a minimal flow disturbance in the central part of the tank.

Polyamide particles (Orgasol) with a mean diameter of 60 μm and a density of 1.020 kg m^−3^ were added to both the salty and the fresh water layers, and to the injected saline solutions to allow optical velocity measurements using the particle image velocimetry (PIV) technique. A 25 W Yag laser operating at a wavelength λ = 532 nm provided a continuous light source. The beam was transmitted through a set of mirrors in the center of the tank where a high-speed rotating mirror enabled to illuminate the full tank over an area of 130 m^2^. The system was allowed to move vertically along a Labview controlled linear axis to scan the whole water depth yielding 12 levels equally spaced by 4 cm, the highest and lowest levels being at 51 cm and 7 cm from the bottom, respectively. Images of 13 m × 9 m were taken with a high-resolution Nikon Camera (D850 SLR, 45MPx) synchronized with the laser system, at a frame rate of 1 Hz. The spatial resolution of 1 mm pixel^−1^ was obtained using an optical lens of 14 mm F2.8 on the camera. Velocity fields were computed using a cross-correlation PIV algorithm encoded with the software UVMAT developed at LEGI. For this purpose, an adaptive multi-pass routine was used, starting with an interrogation window of 35 × 35 pixels and a final window size of 20 × 20 pixels, with a 70% window overlap. Each element of the resulting vector field thus represents an area of roughly 0.5 × 0.5 cm. The velocity vectors were post-processed using a local median filter. The maximum experimental error is estimated to be about 3% in the instantaneous velocity and about 10% in its spatial derivatives.

The velocity and the density at the outlet were monitored by means of an Acoustic Doppler Velocimetry Profiler (ADVP, Vectrino) and a conductivity probe. Both instruments were mounted 1 m downstream of injector S_1_ on a traversing system that enabled continuous measurements along a radial section of 1 m length. In order to investigate the near-bottom dynamics, three velocity components were measured in a vertical section within a 3.5 cm wide bottom layer parallel to the bottom slope, while the density was calculated from conductivity values recorded at a distance of 1 cm from the bottom. An additional ADVP and a conductivity probe were used to capture local velocities and densities 1 cm from the bottom, 4 m downstream of injector S_1_. A conductivity probe was positioned in the central (deep) part of the tank and mounted on a third traverse system enabling to take continuously vertical density profiles.

Initial conditions for the two-layer system were imposed as ρ_top_ = 999.5 kg m^−3^, h_top_ = 36 cm, ρ_bottom_ = 1014.7 kg m^−3^ and h_bottom_ = 21 cm. The rotation period T (referred to as “one day”) was set to 120 s. Injector S_2_ provided a flow of dense water at ρ_2_ = 1010 kg m^−3^ and a flow rate of 0.4 l s^−1^ from the beginning of the experiment. Injector S_1_, positioned 180 degrees downstream from S_2_, provided a flow of dense water at ρ_1_ = 1019.8 kg m^−3^ and a flow rate of 1.6 l s^−1^. Initially, just S_2_ was active: injector S_1_ was switched on 90 minutes later, corresponding to 45 rotation periods (i.e., experimental days); it was then switched off after 180 minutes, when the flow rate of S_2_ was doubled to 0.8 l s^−1^. Then, discharge from S_2_ continued for 60 more minutes. In total, the experiment lasted 270 minutes (i.e., 135 days). Vorticity data from the PIV measurements were normalized by the Coriolis parameter of the tank (10^–1^ s^−1^ – 120 s rotational period) and averaged within the plain deep central area.In the simulation of geophysical phenomena in laboratory tanks, a fundamental point is to achieve a “dynamical similarity” between real-ocean and tank phenomena. In our case, to simulate the Adriatic overflow into the Ionian basin and to reproduce the NIG reversals in the Ionian Sea in the laboratory, two non-dimensional numbers are relevant for the dynamical similarity that need to be achieved.First of all, the Burger number gives the ratio between the Rossby radius of deformation and the geometrical scale of the Ionian basin/the tank for the experiment. Considering the depths in both, the experiments and Ionian basin, the Coriolis parameter f and the density anomaly, expressed using the buoyant acceleration g’, we obtain a similarity between ocean and laboratory phenomena using the experimental values used for the considered experiment. In particular, the combination of those values with an experimental slope of s = 0.1 yields an experimental Burger number like the one observed in the ocean.

Another important similarity that must exist is that between the *in situ* and laboratory ratios of the topographic slope and the initial geostrophic slope, which means that the non-dimensional number g’s/(fV), with V the initial (Adriatic) overflow velocity, has to be preserved in the laboratory experiments. Considering these values for both the Ionian basin and Adriatic outflow and the above-mentioned similarity of the Burger number, we selected the topographic slope of 0.1 in order to fall within the similarity values of the *in situ* conditions ranging from approximately 2.4 to approximately 9.4.

Finally, the experiments also preserved dynamical similarity accounting for frictional effects by considering the Ekman non-dimensional numbers.

### Numerical model

In order to reproduce numerically the laboratory experiment performed in the rotating tank we used a hydrostatic, unsteady nonlinear frontal numerical model^19,34^. Due to a special treatment of movable lateral boundaries, the model is able to accurately reproduce the intrusion of a frontal, bottom as well as intermediate layer in a stratified environment. Moreover, its layered structure allows for the achievement of a very high horizontal resolution (in this case dx = dy = 4 cm) within each layer, with the consequence that small-scale phenomena (in such a case, roll waves and Ekman-like leakage) can also be captured by the model.

### Altimetric data

Altimetric data are the daily Absolute Dynamic Topography (ADT) and the corresponding Absolute Geostrophic Velocities (AGV) projected on the 1/8° Mercator grid distributed by CMEMS-Copernicus Marine Environment Monitoring Service (SEALEVEL_MED_PHY_L4_REP_OBSERVATIONS_008_051). Near surface geostrophic vorticity was averaged over the North Ionian Gyre region (37–39°N, 17–19°E) and normalized by the Coriolis parameter (10^−4^ s^−1^).

### Mean circulation and time scales

To quantify the circulation induced in the upper layer by the injection of dense water at the slope, we consider the equation of conservation of potential vorticity. The mean relative vorticity of the *top* layer over the flat central part of the rotating tank is $\zeta$ = ∂*v*/∂*x* − ∂*u*/∂*y*, with *u* and *v* are the horizontal velocity components along *x* and *y* axes, respectively. If *h* is the time dependent depth of the *bottom* layer, then the variation in depth *dh*/*dt* is related to the injected flow rate *Q* = 1.6 l/s as *dh*/*dt* = *Q*/(π *R*(*t*)^2^), where *R*(*t*) = *R*_0_ + *h*(*t*)/*s* is the radius of the interfacial layer depending on time t because of the sloping boundary with s = *tan ϴ* = *0*.*1*, and *R*_0_ = 2.5 m is the radius of the central (deep) region. The initial thickness of the bottom layer was kept equal to *h*_0_ = 0.21 m. Therefore, the explicit evolution of the bottom layer thickness *h*(*t*) yields as a cubic root of time (nearly linear function for the considered time interval), which can be substituted in the potential vorticity equation of the top layer

$$\frac{\zeta +f}{H-h}={\rm{const}}.,$$

where *f* is the Coriolis parameter, *H* is the total water column thickness (57 cm) and *h* < *H*. The time evolution of the relative vorticity of the top layer follows

$$\zeta =\frac{\left(\,,f,+,{\zeta }_{0}\right)}{\left(H,-,{h}_{0}\right)}\left\{H-s{R}_{0}\left\{{\left[{\left(1+\frac{{h}_{0}}{s{R}_{0}}\right)}^{3}+\frac{3Qt}{\pi s{R}_{0}^{3}}\right]}^{1/3}-1\right\}\right\}-f.$$

However, for the rate of change of the vorticity of the upper layer the following expression is more convenient

$$\frac{\partial \zeta }{\partial t}=-\,\frac{Q}{\pi }\frac{(\zeta +f)}{{({R}_{0}+\frac{h}{s})}^{2}(H-h)}.$$

These relations reproduce very well the experimental data and match the rate of change of vorticity $\partial \zeta$/$\partial t$, calculated from the velocity measurements, between 0.1% and 6%, as evident from Fig. 4. This is an excellent result considering the various simplifications and neglecting important processes as entrainment and friction due to Ekman leakage. The grey line in Fig. 4b represents the above expression.
